# Molecular mutation profile of *pfcrt* in *Plasmodium falciparum* isolates imported from Africa in Henan province

**DOI:** 10.1186/s12936-016-1306-6

**Published:** 2016-05-10

**Authors:** Rui-min Zhou, Hong-wei Zhang, Cheng-yun Yang, Ying Liu, Yu-ling Zhao, Su-hua Li, Dan Qian, Bian-li Xu

**Affiliations:** Department of Parasite Disease Control and Prevention, Henan Province Center for Disease Control and Prevention, Zhengzhou, 450016 People’s Republic of China

**Keywords:** *Plasmodium falciparum*, *Pfcrt*, Chloroquine resistance (CQR), Imported, Africa, Henan province

## Abstract

**Background:**

Anti-malarial drug resistance is a primary public health problem. Haplotypes of *pfcrt* gene have been implicated to be molecular markers of chloroquine (CQ) resistance. This study aims to explore the prevalence of polymorphisms in *pfcrt* in *Plasmodium falciparum*-infected patients imported from Africa in Henan province.

**Methods:**

Blood samples were collected from 502 patients who were infected with *P. falciparum* returning from Africa in Henan province during 2012–2015. The single nucleotide polymorphisms in *pfcrt* (codons 72–76) were assessed by nested PCR with DNA sequencing and restriction digestion, the haplotype prevalences were also determined.

**Results:**

Four haplotypes coding 72–76 of *pfcrt* were found including CVMNK (wild type), CVIET (mutation type), CVIEK (mutation type), and CV M/I N/E/D/K K/T (mixed type), with 61.95 % (311/502), 33.07 % (166/502), 0.20 % (1/502), and 4.78 % (24/502) prevalence, respectively. Except mixed type, CVIET and CVIEK were the largest proportion of the mutant type in West Africa, accounting for 44.83 % (91/203), followed by East Africa (8/21, 38.10 %), North Africa (4/11, 36.36 %), Central Africa (36/135, 26.67 %), and South Africa (28/132, 21.21 %). There was significant difference among the groups (χ^2^ = 23.78, *P* < 0.05). Mixed type was the largest proportion in North Africa (9.09 %), followed by Central Africa (6.67 %), East Africa (4.76 %), South Africa (4.55 %), and West Africa (3.45 %). There was no significant difference among the groups (χ^2^ = 2.31, *P* > 0.05). The position 72 and 73 of *pfcrt* showed predominance for the wild type with rates of 100 % (502/502).

**Conclusions:**

This study identified four haplotypes of *pfcrt* in *P. falciparum*-infected patients imported from Africa in Henan province. The prevalence of mutations in the *pfcrt* was dropped comparing with other people’s researches. It establishes fundamental data for detection of *P. falciparum* CQR with molecular markers for the imported *P*. *falciparum* in China, and it also provides complementary information of CQR for the malaria endemic countries and assesses the evolution of anti-malarial drug resistance.

## Background

Malaria is an infectious disease that has been present in sub-tropical and temperate countries for much of history. It varies widely in epidemiology and clinical manifestation and is responsible for an estimated 214 million clinical episodes and approximately 438,000 deaths per year, of which approximately 90 % occur in Africa [1]. In 2010, the Ministry of Health in China launched an ‘action plan for malaria elimination’, with the goals of eliminating malaria in the entire country by the end of 2020 [2]. There has been no indigenous malaria case in Henan province since 2012, whereas the imported cases have increased year-by-year, most of which were imported *Plasmodium falciparum* from Africa [3–6].

The variability in the spectrum of malarial diseases is the result of several factors, including the distribution of the two primary species of malaria parasites (*P. falciparum* and *P. vivax*), their levels of susceptibility to anti-malarial drugs, the distribution and efficiency of mosquito vectors, climate and other environmental conditions and the behaviour and level of acquired immunity of the exposed human populations [7]. Due to the lack of an effective vaccine, malaria is currently re-infectious and thus its case management depends solely on anti-malarials [8, 9]. Efficacious anti-malarial medicines are critical to malaria control, and continuous monitoring of their efficacy is needed to inform treatment policies in malaria-endemic countries, and to ensure early detection of, and response to, drug resistance [10].

Chloroquine (CQ) was used to combat malaria in 1940s after the Second World War. Since then, it has been considered to be the drug of choice for the treatment of non-severe, uncomplicated malaria and for chemoprophylaxis [11]. Over the years, CQ has proven to be one of the most successful and important drugs ever deployed against malaria, especially in the highly endemic areas of Africa [12]. The tremendous success of CQ and its heavy use for almost 12 years [13] led to the development of resistance in *P. falciparum* during the late 1950s [14–18]. CQ resistance (CQR) was reported for the first time at the Thailand-Cambodia border in 1957 and the Venezuela-Colombia border in 1959 and eventually spread to other countries throughout the world [19–21]. Soon after, CQR *P. falciparum* isolates were found to be widespread in malaria-endemic zones, the mutagenic basis of CQR was made evident by several clinical and epidemiological studies [22, 23]. Amino acid polymorphisms have been found in exon 2 of the *pfcrt* gene at residues 72, 74, 75 and 76 in *P. falciparum* isolates, suggesting that they may be involved in the genetic characterization of CQR and CQ sensitivity (CQS). Accordingly, whereas the C_72_ V_73_M_74_ N_75_ K_76_ haplotype is considered to be CQS, parasites with polymorphisms at any of these amino acid positions are considered to be CQR [24, 25]. In this study, the prevalence of polymorphisms in *pfcrt* gene were determined from imported *P. falciparum* patients in Henan province who came back from Africa.

## Methods

### Sample collection and DNA extraction

Blood samples were collected from patients with *P. falciparum* infection prior to treatment. All of the patients came from Africa in year 2012–2015. The final diagnosis was made by microscopic examination of Giemsa-stained thick blood films and nested PCR. For each sample, approximately 200 μl of finger-prick blood was spotted on a piece of Whatman 3 M 903 filter paper and air-dried. The dried filters were stored in individual plastic bags at −20 °C until DNA extraction. Parasite DNA was extracted from the blood filters using a QIAamp DNA mini kit (Qiagen, Valencia, CA, USA) following the manufacturer’s instruction.

### Nested PCR amplification and DNA sequencing

Nested PCR [26–28] was performed to amplify the fragments of *pfcrt* using a DNA thermal cycler (Mastercycler nexus, Eppendorf Ltd, Germany). The primary PCR was performed in a 20-μl reaction volume containing 6.0 μl ddH_2_O, 1.0 μl each of OuterP1, and OuterP2 primers (10 μmol/l), 10 μl 2 × Go Taq Green Master Mix (Promega, USA) and 2.0 μl DNA template. The PCR reaction condition was 94 °C for 3 min; 35 cycles of 94 °C for 30 s, 56 °C for 30 s, and 60 °C for 60 s; and a final extension at 60 °C for 5 min. The nested PCR contained 7.0 μl ddH_2_O, 1.0 μl each of InnerP1, and InnerP2 primers (10 μmol/l), 10 μl 2 × Go Taq Green Master Mix, 1.0 μl from the first PCR product. The PCR reaction condition was the following: 94 °C for 3 min; 35 cycles of 94 °C for 30 s, 48 °C for 30 s, and 65 °C for 60 s; and a final extension at 65 °C for 5 min. The amplified products were purified from an agarose gel and sequenced by Sangon Biotech Co Ltd (Shanghai, China). Primers were also synthesized by Sangon Biotech Co Ltd. Primer sequences are provided in Table 1.

**Table 1:** PCR primer sequences used for the amplification sequence encoding *pfcrt*

Gene	Primer	Sequence (5′–3′)	Size (bp)
*pfcrt*	OuterP1	CCGTTAATAATAAATACACGCAG	537
	OuterP2	CGGATGTTACAAAACTATAGTTACC	
	InnerP1	TGTGCTCATGTGTTTAAACTT	145
	InnerP2	CAAAACTATAGTTACCAATTTTG	

### Restriction digest of *pfcrt* gene amplicons

Enzymatic digestion of the resulting 145 base pair fragment of the *pfcrt* amplicons was done using Apo I restriction enzyme (Fermentas Life Sciences) according to the manufacturer’s instructions.

### Sequencing alignments and data analysis

Sequence alignments and analysis were carried out using BioEdit software. Amino acid sequences were compared with 3D7 strain, which was obtained from the Chinese Centre for Disease Control and Prevention. The sequences of the amplicons were aligned with 3D7 strain published data from the NCBI database by BLAST analysis. All the data were proceed using Excel to build database and the SPSS 17.0 software was used for analysis. The Person’s Chi square test was used to determine significance of results. A *P* value <0.05 was considered statistically significant.

### Ethical approval

The study was reviewed and approved by the Project of Medical Science and Technology of Henan province (No. 201304053). Before sampling, the purpose was illustrated to participants and consent was obtained from those who agreed to participate in the survey.

## Results

### General characteristics of patients

Blood samples were collected from 502 patients who were infected with *P. falciparum* returning from 26 countries of Africa to Henan province during 2012–2015. The male:female ratio was 70.71:1 (495/7). The mean age was 38.07 ± 9.31 years (range 17–70 years old), of which only 2 patients were older than 60 years and one patient was under 18 years; 502 patients were returned from 26 countries in Africa, the majority (40.44 %, 203) of whom were from West Africa, followed by Central Africa (26.89 %, 135) and South Africa (26.29 %, 132). The minority were returned from East Africa and North Africa, accounting for 4.18 % (21) and 2.19 % (11), respectively.

### PCR–RFLP of the *pfcrt* gene

**Fig. 1:**
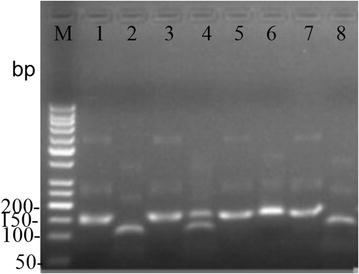
Identification of the four haplotypes by enzyme digestion. *Lanes* *1*, *3*, *5* and *7* were the nested PCR product of the four haplotypes. *Lanes* *2*, *4*, *6* and *8* were digested by Apo I. M, molecular marker; *lanes* *1* and *2*, CVMNK; *lanes* *3* and *4*, CVM/I N/E/D/K T/K; *lanes* *5* and *6*, CVIET; *lanes* *7* and *8*, CVIEK

**Fig. 2:**
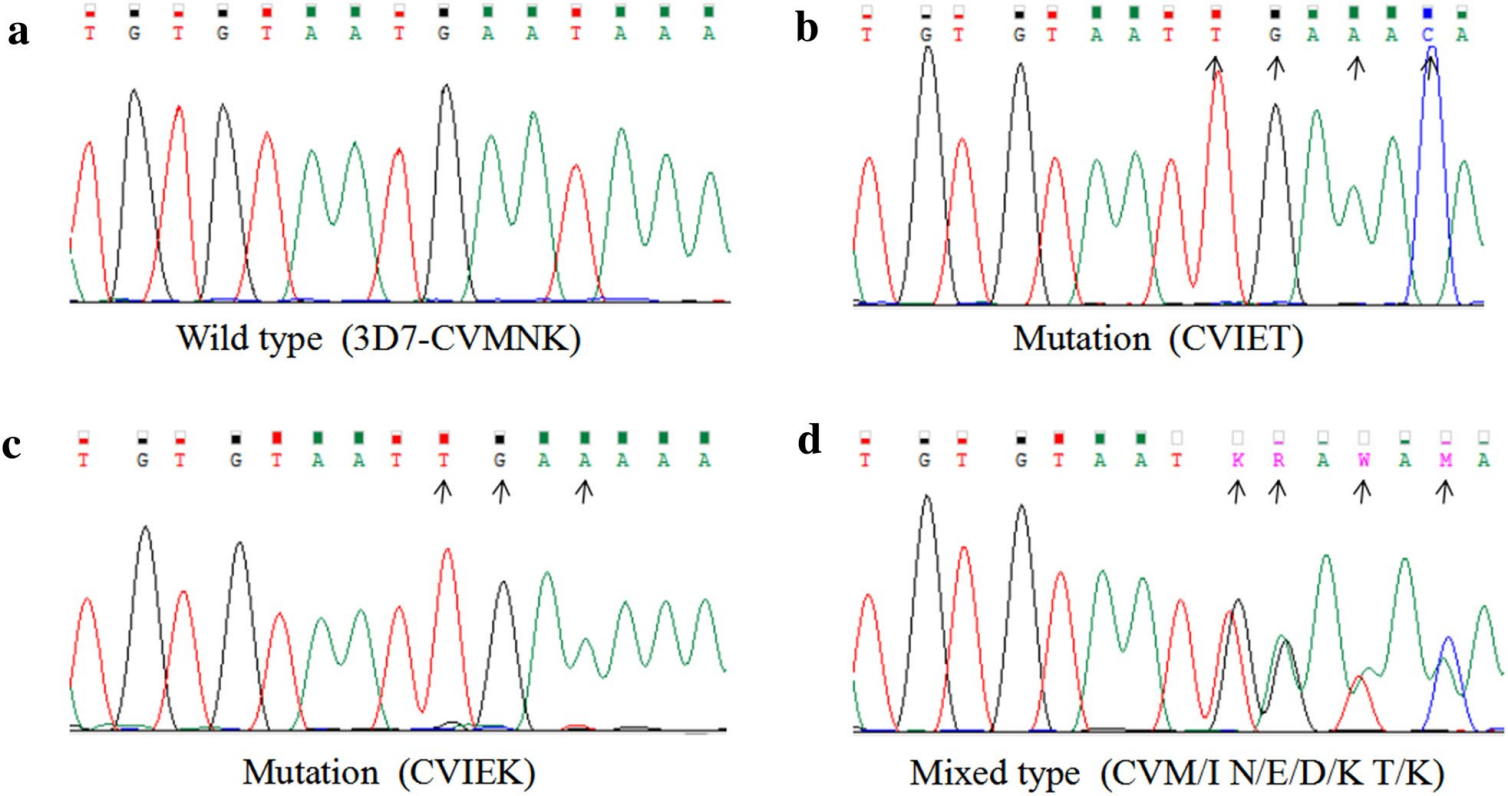
Sequencing profile of nested PCR product of *pfcrt* genotypes detected in *Plasmodium falciparum* isolates imported from Africa in Henan province. **a** The wild type. The haplotype named CVMNK was got from 3D7. **b** and **c** The mutation. The haplotype was named CVIET and CVIEK, respectively. **d** The mixed type. The position was showed by degenerate base. The haplotype was named CVM/I N/E/D/K T/K. The mutation position is indicated by the *arrow*

The nested PCR yielded a 145-bp PCR product for each of the 502 samples. These products contained the codons of interest, 72 through 76, which were analysed both by enzymatic digestion and sequencing. The resulting of Apo I enzymatic digestion yielded two fragments of 31 and 114 bp in the case of the parasite strains containing the wild K76 variant, whereas the mutant T76 variant yielded only one fragment since there was no digestion. Multi-clonal variants yielded three fragments (145, 114 and 31-bp products). A photo of agarose gel electrophoresis is shown in Fig. 1. Restriction digest of the 502 samples gave the following results: 166 (33.07 %) mutant types, 312 (62.15 %) wild types and 24 (4.78 %) mixed infections, which were also confirmed by sequencing (see Fig. 2).

### *Pfcrt* mutations and haplotypes

A 145-bp fragment of the *pfcrt* gene, including residues 72–76, was successfully sequenced from all the samples. The position 72 and 73 of *pfcrt* were all the wild type with rates of 100 % (502/502). Four haplotypes coding 72–76 of *pfcrt* were found, including CVMNK (wild type), CVIET (mutation type), CVIEK (mutation type), and CV M/I N/E/D/K K/T (mixed type), with 61.95 % (311/502), 33.07 % (166/502), 0.20 % (1/502) and 4.78 % (24/502) prevalence, respectively. The detail information and sequencing results of the mutations are shown in Fig. 2 and Table 2, respectively.

**Table 2:** Distribution of the CQR-*pfcrt* haplotypes from Africa-imported cases

Region	Country	Total	CVMNK	CVIET	CV M/I N/E/D/K K/T
North Africa		11	6 (54.55 %)	4 (36.36 %)	1 (9.09 %)
	Sudan	9	6 (66.67 %)	3 (33.33 %)	0 (0.00 %)
	Libya	2	0 (0.00 %)	1 (50.00 %)	1 (50.00 %)
East Africa		21	12 (57.14 %)	8 (38.10 %)	1 (4.76 %)
	Ethiopia	2	0 (0.00 %)	2 (100 %)	0 (0.00 %)
	Kenya	3	3 (100 %)	0 (0.00 %)	0 (0.00 %)
	Tanzania	10	6 (60.00 %)	3 (30.00 %)	1 (10.00 %)
	Uganda	6	3 (50.00 %)	3 (50.00 %)	0 (0.00 %)
South Africa		132	98 (74.24 %)	28 (21.21 %)	6 (4.55 %)
	Angola	101	69 (68.32 %)	26 (25.74 %)	6 (5.94 %)
	Zimbabwe	1	1 (100 %)	0 (0.00 %)	0 (0.00 %)
	Mozambique	10	9 (90.00 %)	1 (10.00 %)	0 (0.00 %)
	Zambia	20	19 (95.00 %)	1 (5.00 %)	0 (0.00 %)
West Africa		203 (202 + 1^a^)	105 (51.72 %)	90 (44.33 %)	7 (3.45 %)
	Benin	5	1 (20.00 %)	4 (80.00 %)	0 (0.00 %)
	Togo	5	4 (80.00 %)	1 (20.00 %)	0 (0.00 %)
	The Republic of Guinea	33	21 (63.64 %)	11 (33.33 %)	1 (3.03 %)
	Ghana	23	19 (82.61 %)	3 (13.04 %)	1 (4.35 %)
	Ivory Coas	10	7 (70.00 %)	3 (30.00 %)	0 (0.00 %)
	Liberia	23	1 (4.35 %)	22 (95.65 %)	0 (0.00 %)
	Mali	4	2 (50.00 %)	1 (25.00 %)	1 (25.00 %)
	Nigeria	74 (73 + 1^a^)	40 (54.05 %)	31 (41.89 %)	2 (2.7 %)
	Sierra Leone	25	9 (36.00 %)	14 (56.00 %)	2 (8.00 %)
	Burkina Faso	1	1 (100 %)	0 (0.00 %)	0 (0.00 %)
Central Africa		135	90 (66.67 %)	36 (26.67 %)	9 (6.67 %)
	Equatorial Guinea	75	58 (77.33 %)	11 (14.67 %)	6 (8.00 %)
	Congo	23	11 (47.83 %)	12 (52.17 %)	0 (0.00 %)
	Gabon	6	1 (16.67 %)	5 (83.33 %)	0 (0.00 %)
	Cameroon	23	14 (60.87 %)	6 (26.09 %)	3 (13.04 %)
	Chad	7	5 (71.43 %)	2 (28.57 %)	0 (0.00 %)
	Central Africa Republic	1	1 (100 %)	0 (0.00 %)	0 (0.00 %)

^a^There was only one *P. falciparum* isolate from Nigeria with CVIEK haplotype

### Distribution of the CQR-*pfcrt* haplotypes

Four haplotypes coding 72–76 of *pfcrt* were found; only one patient harboured the CVIEK, who was returned from Nigeria in West Africa. The other three haplotypes were found in the every region of Africa; 203 isolates returned from ten countries in West Africa carried four haplotypes: CVMNK, CVIET, CVIEK and CV M/I N/E/D/K K/T, with 51.72 % (105/203), 44.33 % (90/203), 0.49 % (1/203) and 3.45 % (7/203) prevalence, respectively. 135 isolates returned from six countries in Central Africa carried three haplotypes: CVMNK, CVIET and CV M/I N/E/D/K K/T, with 66.67 % (90/135), 26.67 % (36/136) and 6.67 % (9/135) prevalence, respectively. A total of 132 isolates returned from four countries in South Africa carried three haplotypes: CVMNK, CVIET and CV M/I N/E/D/K K/T, with 74.24 % (98/132), 21.21 % (28/132) and 4.55 % (6/132) prevalence, respectively. Twenty-one isolates returned from four countries in East Africa carried three haplotypes: CVMNK, CVIET and CV M/I N/E/D/K K/T, with 57.14 % (12/21), 38.10 % (8/21) and 4.76 % (1/21) prevalence, respectively. Eleven isolates returned from two countries in North Africa carried three haplotypes: CVMNK, CVIET and CV M/I N/E/D/K K/T, with 54.55 % (6/11), 36.36 % (4/11) and 9.09 % (1/11) prevalence, respectively. Except mixed type, CVIET and was the largest proportion of the mutant type in West Africa, accounting for 44.33 % (90/203), followed by East Africa (8/21, 38.10 %), North Africa (4/11, 36.36 %), Central Africa (36/135, 26.67 %) and South Africa (28/132, 21.21 %). There was significant difference among the groups (χ^2^ = 23.78, *P* *<* 0.05). There was the largest proportion of the mixed type in North Africa (9.09 %), followed by Central Africa (6.67 %), East Africa (4.76 %), South Africa (4.55 %) and West Africa (3.45 %). There was no significant difference among the groups (χ^2^ = 2.31, *P* > 0.05) (see Table 2).

## Discussion

Information on the distribution and patterns of anti-malarial drug resistance is essential for implementation of effective malaria control programmes and disease surveillance in all malaria-endemic areas of the world. Early detection of occurrence and spreading of anti-malarial drug resistance would be greatly enhanced by the application of valid anti-malarial resistance molecular markers. CQ has been used widely in African regions for several decades and formed a strong selection pressure on parasite populations. The World Health Organization advocated a complete ban on artemisinin monotherapy for uncomplicated malaria in 2006 [29]. The national drug policy of China was updated in 2009, and first-line drugs currently used to treat falciparum malaria are ACT, which includes dihydroartemisinin-piperaquine (DHA-PIP), artesunate+amodiaquine, artemisinin-naphthoquine phosphate and artemisinin-piperaquine [30]. Among the candidate genes investigated to date, *pfcrt* mutation is widely acceptable as a reliable marker of CQR in *P. falciparum* in epidemiological studies [23, 31]. So it is important to understand the molecular mutation profiles of *P. falciparum* parasite for anti-malarial drug resistance and use this foundational information for detection of molecular pattern.

For CQR *P. falciparum*, two principal haplotypes, with the amino acid sequences C_72_ V_73_ I_74_ E_75_ T_76_ and S_72_ V_73_ M_74_ N_75_ T_76_ [24, 25] are widely distributed. Due to the widespread yet structured present-day distribution across *P. falciparum*-endemic zones across the globe, these two haplotypes are hypothetically considered to be CQR mother haplotypes and the other minor haplotypes are believed to have been derived from them [24, 25]. It has been established that CVIET and SVMNT are widely prevalent; whether all of the other minor haplotypes were derived from these two or evolved independently is still an open question.

Mutations in *pfcrt* are connected to resistance of anti-malarial medication, including CQ, amodiaquine (AQ) and lumefantrine (LU), and the haplotypes of *pfcrt* CVIET and SVMNT have been implicated [32]. The *pfcrt* CQR haplotypic view in Africa is completely biased towards the CVIET haplotype, owing to the wide usage of CQ and AQ drugs in many African countries [33]. This study indicated 33.07 % isolates harboured the *pfcrt* mutant CVIET, while 4.98 % isolates had the CVIET-derived haplotypes, CV M/I N/E/D/K K/T (4.78 %) and CVIEK (0.20 %). The two main methods, namely Apo I enzymatic digestion and the sequencing, employed here to analyse the *pfcrt* gene status of the parasite strains from the study participants served to confirm or complement findings from each of the methods. There was no SVMNT haplotype found in isolates returned from Africa in this study. The *pfcrt* SVMNT is more commonly found in South America and some Asian countries [34], but rarely reported from the African continent. The result in this study that no SVMNT haplotype found in isolates returned from Africa confirm the above conclusion. Except mixed type, there was the largest proportion of the mutant type in West Africa, accounting for 44.83 % (CVIET and CVIEK, 91/203); this difference might come from the amount of AQ used in different regions. In this study, 4.78 % mixed type (wild type and mutant type) of *pfcrt* was detected in *falciparum* malaria isolates returned from ten countries (Angola, Equatorial Guinea, Cameroon, Nigeria, Sierra Leone, The Republic of Guinea, Ghana, Mali, Libya, Tanzania). It was reported that 6.62 % mixed type of *pfcrt* was found in malaria isolates from Bioko Island, Equatorial Guinea [35], which was not reported in neighbouring countries [36–39]. This study enriched the distribution of the mixed type of *pfcrt*. The CVIEK haplotype was reported to be detected in Central Africa Republic, Gabon, Sudan, Thailand, and Eastern India [40–46]. In this study, among 502 isolates, only one patient harboured the CVIEK haplotype, who returned from Nigeria. It was the first report that CVIEK haplotype found in West Africa.

Five-hundred and two patients were returned from 26 countries in Africa; half of them (250/502, 49.80 %) were from three countries: Angola (101), Equatorial Guinea (75) and Nigeria (74). The remainder returned from the other 23 countries, but there were 16 countries from which fewer than or equal to ten patients returned, so it was hard to fully explain the situation of the *pfcrt* in each country.

In regions with high malaria endemic, the withdrawal of CQ as first-line treatment of *P. falciparum* infections has typically led to the restoration of CQ susceptibility through the reexpansion of the wild type allele of pfcrt at the expense of less fit mutant alleles carrying the CQR marker. In French Guiana, despite the fixation of the K76T allele, the prevalence of CQR isolates progressively dropped from >90 % to <30 % during 17 years after CQ withdrawal in 1995 [47]. In this study, the prevalence of CVIET haplotype among 502 isolates was 33.07 %, which was consistent with the above results. Gharbi et al. reconfirmed this conclusion, they carried out a longitudinal study assessing the return of chloroquine susceptibility of *P. falciparum* in isolates from travellers returning from West and Central Africa, during 2000–2011, the prevalence of the fcrt76T mutant genotype significantly decreased [48, 49].

Travelers who return from endemic countries infected with malaria often present with low immunity against the parasites and there is no risk of re-infection, so they are a particularly valuable source of information. Many settings in endemic countries lack the financial resources necessary to maintain a sustainable, accurate and reliable anti-malarial resistance surveillance system, resulting in gaps in the spatial and temporal available information. This study can provide complementary information of CQR for the malaria endemic countries.

## Conclusions

This study reports the prevalence of polymorphisms in *pfcrt* gene in blood sample obtained from *P. falciparum*-infected patients imported from Africa in Henan province, and it illustrates the situation of CQR for the patients of *P. falciparum* imported from Africa, which is essential for implementation of effective malaria elimination programmes and drug resistance surveillance in Henan province. Continuous molecular surveillance with *pfcrt* gene of imported *P. falciparum* as an anti-malarial drug resistance marker is exceedingly recommended in China. It will be useful for the malaria endemic countries to assess the evolution of anti-malarial drug resistance and provide complementary information of CQR.
